# A cost-effective Co_3_O_4_@WO_3_ hetero-structure derived from WO_3_@Co-CoPBA for oxygen evolution reaction

**DOI:** 10.1039/d5ra04599a

**Published:** 2025-08-22

**Authors:** Zhenwei Yan, Zihao Wei, Zhaojun Tan, Shuaihui Guo, Zhipeng Fang, Wen Wang, Gang Li, Xianjie Yuan, Mingqi Tang, Zaiqiang Feng

**Affiliations:** a School of Mechanical Engineering, North China University of Water Resources and Electric Power Zhengzhou 450011 PR China yanzhenwei@163.com; b School of Materials Science and Engineering, North China University of Water Resources and Electric Power Zhengzhou 450011 PR China

## Abstract

The oxygen evolution reaction (OER) is hindered by the sluggish kinetics, high costs, and poor stability of noble metal catalysts (*e.g.*, RuO_2_), as well as low atomic utilization and limited accessibility of active sites in transition metal oxide catalysts. To address these challenges, this study develops a core–shell structured WO_3_@Co-CoPBA heterostructure as an efficient OER electrocatalyst. Co-CoPBA nanocubes are hydrothermally synthesized and then loaded with WO_3_ nanorods, followed by gradient annealing under N_2_ atmosphere (optimized at 500 °C) to form a Co_3_O_4_@WO_3_ heterojunction. Characterization and electrochemical evaluations reveal that annealing at 500 °C induces topological reconstruction of Co-CoPBA into porous Co_3_O_4_ and graphene cores, Co sites in Co_3_O_4_ serve as the catalytic active centers, forming a strong electronic coupling interface with the WO_3_ shell. This architecture significantly enhances the density of active sites (electrochemically active surface area of 3.8 cm^2^) and charge transfer efficiency (Tafel slope of 55.12 mV dec^−1^). The catalyst delivers an overpotential of 315 mV at 100 mA cm^−2^ in 1 M KOH, outperforming commercial benchmark catalyst RuO_2_ (372 mV). It exhibits exceptional stability with almost no performance decay after 100 h. Density functional theory (DFT) calculations demonstrate that interfacial electronic restructuring modulates the d-band center, optimizing the adsorption Gibbs free energy of OOH* intermediates and thereby improving intrinsic catalytic activity. This work provides an effective interface engineering strategy for designing high-performance, low-cost transition metal-based electrocatalysts.

## Abstract 2

A simple and novel Co_3_O_4_@WO_3_ hetero-structured heterostructures with cost-effective and high-performance alkaline OER activity was synthesize through hydrothermal synthesis of WO_3_ on Co-CoPBA precursors coupled with annealing temperature modulation.

## Graphical abstract

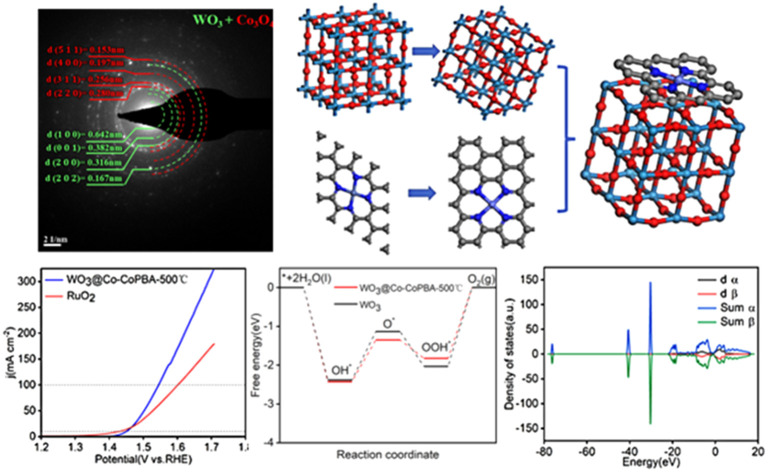

## 1 Introduction

Driven by global energy transition and carbon neutrality goals, the development of sustainable energy technologies has become imperative. Hydrogen energy, with its high-energy density (120 MJ kg^−1^) and zero carbon emissions (with water as the only combustion product), has been widely recognized by the International Energy Agency (IEA) as a promising clean energy carrier.^1^ The water electrolysis technology for hydrogen production, powered by fluctuating renewable energy sources, has garnered significant attention due to its advantages of being both clean and efficient, as well as the convenience of hydrogen purification^2^. However, the sluggish kinetics of oxygen evolution reaction (OER) and hydrogen evolution reaction (HER), along with the high overpotentials, result in low energy conversion efficiency. Developing efficient and stable catalysts is the key to overcoming this bottleneck. Although noble metal oxide RuO_2_ is still regarded as the benchmark catalyst for OER owing to its superior catalytic performance, its scarcity, high cost, and stability issues limit its scalability.^3–5^

Transition metal-based materials, such as those based on Ni, Co, Mn and Fe, and their oxides and hydroxides doped with non-metallic elements (N, O, S, P) have become research hotspots for non-precious metal electrocatalysts, which demonstrate environmental sustainability and cost-effectiveness in large-scale applications.^6–8^ However, their active sites are primarily concentrated in the surface/edge regions, resulting in low atomic utilization in the bulk phase. These limitations result in limited spatial availability of active sites and mass transport barriers in the bulk phase, causing a decline in the mass activity and overall performance of the catalyst.^9^ Layered WO_3_ crystals exhibit advantages in HER/OER bifunctional catalysis due to their intrinsically high conductivity and electrochemical stability.^10–15^ The nanorod array structure enhances adsorption capacity and improves catalytic kinetics through a high specific surface area, while the heterostructure with TiO_2_/ZnO/α-Fe_2_O_3_ can achieve performance enhancement *via* interfacial charge synergism.^16–19^ WO_3_ is inert to inorganic acids except for HF, making it suitable for acidic electrolysis environments. However, the irreversible reaction between OER intermediates and the lattice generates soluble peroxotungstate, resulting in a decrease in the stability of the catalytic system.^20,21^

Metal–organic frameworks (MOFs) have emerged as exemplary precursors for electrocatalysts owing to their tunable structural and compositional flexibility, ultrahigh specific surface areas, and open-framework architectures. These characteristics render them particularly suitable for applications in ion intercalation energy storage and catalysis.^22–24^ Among their subclasses, Prussian blue analogues (PBAs) exhibit unique advantages for multi-ion channel systems through cyano-bridged structures that enable reversible metal-ion insertion.^25–30^ These materials feature three-dimensional open frameworks with high active site exposure, rapid charge-transfer kinetics, and multiple ionic conduction pathways. However, their practical applications are hindered by intrinsically low electrical conductivity and limited accessibility of surface active sites, which collectively impede charge transport and cause sluggish reaction kinetics.^31^

Although several Co-based MOF/metal oxide heterostructures have been reported for OER applications, they typically suffer from poor interfacial coupling, limited active site accessibility, and inadequate long-term stability under alkaline conditions. Most existing approaches rely on physical mixing or conventional growth methods that fail to establish strong electronic interactions between components. Furthermore, the dissolution of cobalt species in alkaline electrolytes remains a critical challenge that limits their practical applicability. Therefore, developing novel interface engineering strategies that can simultaneously optimize electronic coupling, enhance stability, and maximize active site utilization represents a significant advancement in the field.

Based on these challenges, our design strategy was motivated by addressing the complementary limitations of individual components through synergistic heterostructure engineering. While WO_3_ exhibits high conductivity and electrochemical stability, it suffers from limited active sites and poor OER kinetics, conversely, Co-CoPBA provides abundant active sites and high cobalt content but lacks electrical conductivity and charge transfer efficiency. The core–shell architecture was specifically designed to maximize these complementary advantages: the WO_3_ shell serves as a conductive framework and provides structural protection, while the Co-CoPBA core delivers high-density active sites, and the heterointerface enables electronic coupling that modulates the d-band center and optimizes intermediate adsorption energies according to the Sabatier principle and the d-band theory.

In this work, we rationally designed a core–shell-structured WO_3_@Co-CoPBA heterostructure through synergistic integration, where Co sites serve as the primary active centers while WO_3_ functions as an electronic modulator and structural stabilizer. The preparation method involves hydrothermal fabrication of Co-CoPBA nanocubes followed by WO_3_ nanorod loading, ultimately forming a single core–double shell WO_3_@Co-CoPBA heterojunction after annealing at different high temperatures under N_2_ atmosphere. The above-mentioned materials possess two advantageous features: (1) the synergistic coupling of WO_3_ components and graphene layers significantly enhances the density of active sites and (2) the strong electronic coupling at the heterointerface simultaneously suppresses active material dissolution and reinforces structural stability. The WO_3_@Co-CoPBA reveals remarkable OER performance with substantially reduced overpotentials compared to commercial RuO_2_ benchmarks. Density functional theory (DFT) calculations further elucidate that the interfacial electronic reconstruction modulates Gibbs free energy of HER/OER intermediates, while d-band center engineering of the catalyst strengthens electronic coupling between active sites and OOH*, enhancing molecular adsorption capabilities. This study provides novel insights into the interface engineering design of transition metal-based electrocatalysts for energy conversion systems.

## 2 Experimental section

### 2.1 Chemicals

Potassium hexacyanocobaltate (K_3_[Co(CN)_6_], 1.0 g, 99.0%), cobalt nitrate hexahydrate (Co(NO_3_)_2_·6H_2_O, 2.0 g, 98.5%), sodium citrate (C_6_H_5_Na_3_O_7_, 0.5 g, 99.0%), sodium tungstate (Na_2_WO_4_, 0.8 g, 99.5%), sodium sulfate (Na_2_SO_4_, 1.2 g, 99.0%), hydrochloric acid (HCl, 2 mL, 37% concentration, analytical grade), anhydrous ethanol (C_2_H_6_O, 50 mL, 99.7%), and Nafion solution (20 μL, 5 wt%, Sigma-Aldrich) were all purchased from Aladdin Co., China. All reagents were of analytical grade and were used directly without further purification.

### 2.2 Material preparation

Our design strategy was specifically motivated by addressing the complementary limitations of individual components through synergistic heterostructure engineering. Unlike conventional Co-based MOF approaches that often result in poor electrical coupling, we employed Co-CoPBA as a unique precursor that enables topological reconstruction into porous Co_3_O_4_ cores while simultaneously forming graphene encapsulation layers. This approach fundamentally differs from existing literature by creating triple synergistic effects: (1) WO_3_ nanorods provide high conductivity and structural protection, (2) Co_3_O_4_ cores deliver abundant active sites, and (3) graphene layers enhance electron transport while preventing active material dissolution.

Co-CoPBA nanocubes were synthesized using a simple co-precipitation method. At room temperature, cobalt nitrate hexahydrate (Co(NO_3_)_2_·6H_2_O, 2.0 g) and sodium citrate (C_6_H_5_Na_3_O_7_, 0.5 g) were dissolved in 50 mL of deionized water under magnetic stirring to form solution A. The concentration of Co(NO_3_)_2_ was 0.137 M and the concentration of sodium citrate was 0.034 M. In addition, potassium hexacyanocobaltate (K_3_[Co(CN)_6_], 1.0 g) was dissolved in 30 mL of deionized water to prepare solution B with a concentration of 0.101 M. Then, solution B was slowly added dropwise to solution A under continuous stirring (300 rpm) over 30 minutes to ensure uniform nucleation. The mixed solution was aged at room temperature for 24 hours. The product was collected by centrifugation, washed several times with ethanol and water, and then dried overnight at 60 °C. Na_2_WO_4_·H_2_O and Na_2_SO_4_ were dissolved in deionized water containing HCl, then the resulting solution was transferred to a high-pressure reactor lined with PTFE, and a hydrothermal reaction was performed at 180 °C for 12 h. The resulting white product was washed and dried. The obtained sample was named WO_3_ nanorods. The synthesized Co-CoPBA precursor was mixed with WO_3_ nanorods in an aqueous medium, and hydrothermal treatment was performed at 120 °C for 24 hours. After washing and drying, a pink precursor WO_3_/Co-CoPBA was produced, which ultimately transformed into a black-gray WO_3_@Co-CoPBA heterostructure. After annealing in an Ar atmosphere, heterostructures were formed.

### 2.3 Physicochemical characterization

The morphological and structural characterization of the products was examined using scanning electron microscopy (SEM, ZEISS Sigma 300, Germany, 1–30 kV) and transmission electron microscopy (TEM, FEI Tecnai G2 F20, USA, 200 kV, 0.24 nm) equipped with energy-dispersive spectroscopy (EDS, Oxford INCA). Physicochemical characterization was carried out by X-ray diffraction (XRD, Bruker D8 Focus, Germany) and X-ray photoelectron spectroscopy (XPS, Kratos AXIS Ultra DLD, UK). The electrochemical characterization protocols followed established methodologies for accurate surface analysis.^32^ XRD patterns were collected on a D8 Focus (Bruker) diffractometer with Ni-filtered Cu Kα radiation (*λ* = 1.54 Å). XPS measurements were recorded using a Kratos AXIS Ultra Spectrometer using a focused monochromatized Al Kα radiation (hν = 1486.6 eV).

### 2.4 Electrochemical characterization

The OER activity of the synthesized catalysts was systematically evaluated in a three-electrode configuration using a CHI 770E electrochemical workstation (CH Instruments, USA). A Hg/HgO reference electrode, a platinum foil counter electrode, and a catalyst-loaded foam nickel–iron substrate (10 mm × 10 mm) served as the reference electrode, counter electrode, and working electrode, respectively. The working electrode was prepared by dispersing 20 mg of catalyst powder in a mixed slurry containing 20 μL of 5 wt% Nafion solution, 0.5 mL deionized water, and 0.5 mL absolute ethanol. After sonication to form a uniform suspension, an appropriate volume of the slurry was pipetted onto the foam nickel–iron substrate and air-dried at room temperature to yield a well-adhered electrode structure. All electrochemical measurements were conducted using a reversible hydrogen electrode (RHE) reference system to ensure inter-system comparability and establish a unified potential reference. The measured potentials were converted to the RHE scale using the Nernst equation, *E*(RHE) = *E*(Hg/HgO) + 0.059 × pH + 0.098 V, *E*°(Hg/HgO) = 0.098 V *vs.* RHE at pH14.

The overpotential for OER was calculated using the equation: *η* = *E*(applied) − *E*°(OER) − iR, where *η* represents the overpotential (*V*), *E*(applied) is the applied potential *vs.* RHE (*V*), *E*°(OER) = 1.23 V *vs.* RHE is the thermodynamic potential for oxygen evolution reaction, *i* is the current density (*A*), and *R* represents the uncompensated solution resistance (Ω) determined from EIS measurements. The overpotential values reported in this study represent the additional potential required beyond the thermodynamic requirement to achieve specific current densities during OER.

The OER performance of WO_3_@Co-CoPBA-500 °C and its annealing-temperature-controlled counterparts (300–700 °C) was systematically evaluated in 1 M KOH, alongside commercial RuO_2_. The electrochemical measurements were performed in a three-electrode setup with electrode surface activation *via* 10 cycles of cyclic voltammetry (CV) (10 mV s^−1^, 0.2–0.8 V), followed by linear sweep voltammetry (LSV) measurements at 5 mV s^−1^.

To evaluate the electrochemically active surface area (ECSA), double-layer capacitance (*C*_dl_) was measured *via* CV in the non-faradaic potential region. The effective active area was calculated using the formula ECSA = *C*_dl_/0.042, enabling comparison of active site density across different annealing temperatures. The ECSA determination method is as follows: (1) systematically identify the optimal non-faradaic potential region through CV screening across multiple potential windows; (2) use multiple scan rates for verification to confirm the linear relationship between current and scan rate; (3) verify that pseudocapacitive contributions are minimized within the selected potential range; and (4) conduct reproducibility testing to ensure the reliability of the measurements.

Electrochemical impedance spectroscopy (EIS) technology measures the impedance response of a system by applying an alternating current signal. Its core is to quantitatively obtain the charge transfer resistance (*R*_ct_), which reflects the reaction kinetics and is an important indicator for evaluating catalyst activity.

### 2.5 DFT simulations

Density functional theory (DFT) calculations play a pivotal role in elucidating reaction mechanisms, predicting active sites, optimizing catalyst structures, and analyzing electron transfer kinetics in OER studies.

While our DFT calculations provide valuable insights into electronic structure and d-band center modulation, experimental techniques such as Mott–Schottky analysis would complement these theoretical predictions by determining flatband potentials, carrier densities, and definitively classifying the heterojunction type (type I, II, or III). Such experimental validation would strengthen the mechanistic understanding of charge separation and transfer processes at the heterointerface.

Density of states (DOS) analysis is an electronic structure characterization method based on DFT calculations. By comparing the distribution of electronic states, the position of the d-band center relative to the Fermi level can be determined, thereby revealing the microscopic mechanism of catalytic performance improvement.

In this work, a Co_3_O_4_@WO_3_ molecular model was constructed using the Materials Studio software with the PBE functional. The Gibbs free energy (Δ*G*) of adsorbed intermediates was calculated *via* the formula: Δ*G* = Δ*E* + Δ*ZPE* − *T*Δ*S* + e*U*.where Δ*E*, Δ*ZPE*, and Δ*S* represent electronic energy, zero-point energy correction, and entropy change, respectively, while e*U* accounts for the applied potential.

The interface engineering approach demonstrated in this work provides a novel theoretical framework for designing high-performance electrocatalysts. By modulating the d-band center position through heterointerface formation, we achieved optimal binding energies for OER intermediates according to the Sabatier principle.

## 3 Result and discussion

**Fig. 1:**
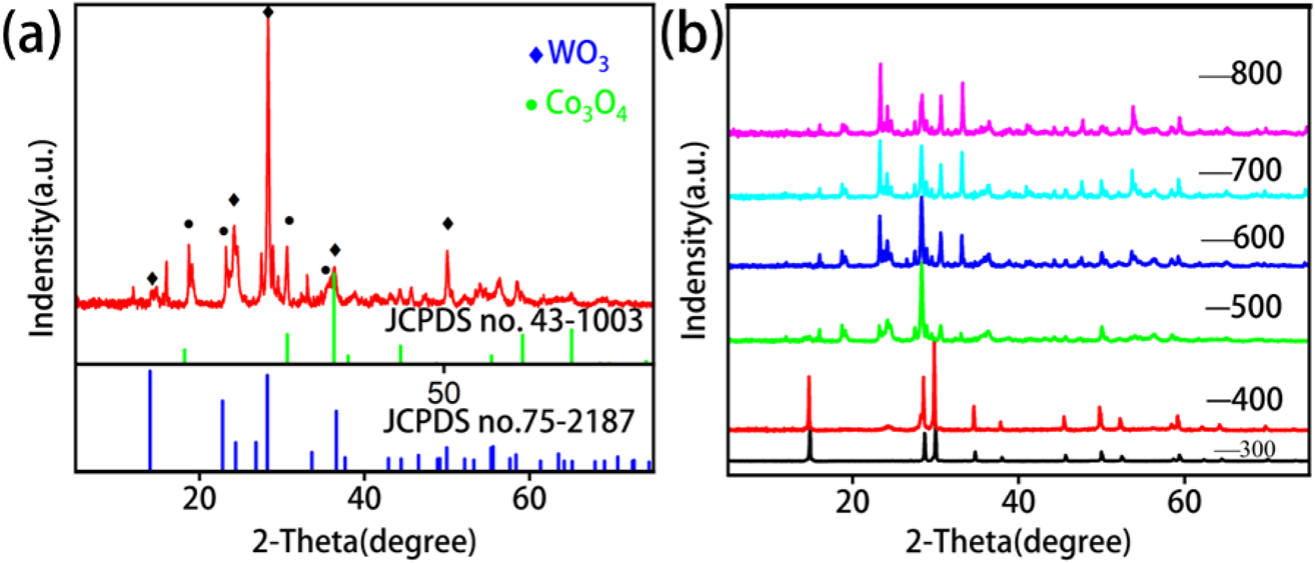
(a) XRD pattern of WO_3_@Co-CoPBA-500 °C; (b) comparative XRD patterns of WO_3_@Co-CoPBA at 300–800 °C.

XRD analysis revealed that the WO_3_@Co-CoPBA heterostructure underwent topological reconstruction at 500 °C, forming a graphene-encapsulated Co_3_O_4_@WO_3_ heterostructure.^33^ The characteristic diffraction peaks at 2*θ* = 13.9°, 23.2°, 24.3°, 26.9°, 28.1°, 33.8°, 36.7°, 37.5°, 51.9°, 61.9° and 65.7°, corresponding to (100), (002), (110), (111), (202), (210), (310), (214) and (304) planes of WO_3_, and three characteristic diffraction peaks of the (111), (220) and (311) lattice planes of Co_3_O_4_, at approximately 2*θ* = 19.0°, 31.2° and 36.8° are observed. The peak for the (003) planes at 2*θ* = 26° unambiguously confirms the presence of graphitic carbon layers (Fig. 1a), confirmed the existence of heterogeneous phase structure. The preservation of distinct, unshifted XRD peaks for both WO_3_ and Co_3_O_4_ phases provides strong evidence against significant tungsten doping in the Co-CoPBA framework. If tungsten incorporation had occurred during the hydrothermal synthesis, characteristic peak shifts, broadening, or new mixed-metal reflections would be expected in the diffraction pattern. The sharp, well-defined peaks at their standard crystallographic positions confirm the formation of a heterostructure composed of distinct phases rather than a tungsten-doped single phase. Comparative XRD patterns at varying annealing temperatures demonstrate consistent peak positions but significantly enhanced intensities with increasing temperature (Fig. 1b), indicating that thermal treatment effectively modulates crystallinity and grain size. This structural evolution governs the exposure of active sites and charge transfer kinetics, ultimately influencing the OER performance.

SEM characterization reveals that Co-CoPBA exhibits a well-defined cubic morphology with sharp edges and approximately 200 nm in size, as shown in Fig. S1a, while WO_3_ nanorod arrays showed uniform alignment with lengths of 5 μm and diameters of 20 nm (Fig. S1b). The heterostructure material WO_3_@Co-CoPBA maintains a 1.5 μm scale (Fig. S1c), where WO_3_ nanorods were homogeneously anchored on the Co-CoPBA surface. The cubic edges exhibit surface roughening due to chemical etching and anion exchange processes, confirming the structural reconstruction. Post-annealing at 500 °C (Fig. S1d) preserves the WO_3_ coating integrity, while Co-CoPBA transforms into a porous Co_3_O_4_ architecture encapsulated by wrinkled graphitic carbon layers. This hierarchical structure provided abundant active sites and high specific surface area, thereby enhancing water-splitting catalytic performance.

In addition, statistical analysis was conducted on the core–shell morphology of WO_3_@Co-CoPBA heterostructures (Fig. S2). Statistical data show that the core diameter exhibits good size uniformity, with an average diameter of 200.6 ± 18.3 nm (160–237 nm, 9.1%) (Fig. S2a). The average shell thickness is 51.3 ± 8.8 nm (34–70 nm. 17.2%) (Fig. S2b). The relatively small coefficients of variation indicate that the synthesis method can effectively control the material morphology and ensure the reproducibility of catalyst performance. Meanwhile, this core–shell heterostructure provides an ideal spatial framework for the synergistic effects of interfacial charge transfer and active sites, which is of great significance for enhancing electrocatalytic performance.

**Fig. 2:**
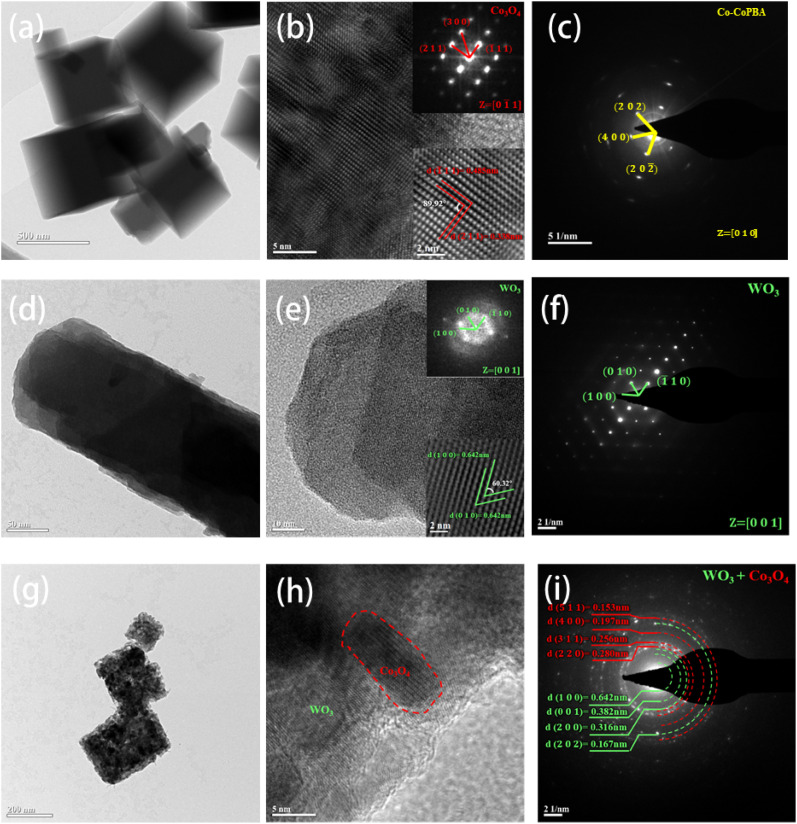
(a) TEM images of Co-CoPBA; (b) high-resolution images; (c) electron diffraction images; (d) TEM images of WO_3_; (e) high-resolution images; (f) electron diffraction images; (g) TEM images of WO_3_@Co-CoPBA-500 °C; (h) high-resolution images; (i) electron diffraction pattern.

TEM characterization of WO_3_, Co-CoPBA precursor, and the WO_3_@Co-CoPBA heterostructure after calcination at 500 °C was performed, elucidating their structural morphology, crystallinity, and interfacial interactions (Fig. 2). The Co-CoPBA precursor (Fig. 2a) exhibits a well-defined cubic morphology with a particle size of 500 nm. HRTEM imaging (Fig. 2b) reveals highly ordered lattice fringes with interplanar spacings of *d* = 0.338 nm (Co_3_O_4_(211)) and *d* = 0.458 nm (Co_3_O_4_(111)). The corresponding Selected area electron diffraction (SAED) pattern (Fig. 2c) displays ring-like diffraction features, confirming the single-crystalline nature of Co-CoPBA. For WO_3_ (Fig. 2d), TEM images show a typical nanorod morphology with a diameter of 20 nm. HRTEM (Fig. 2e) reveals uniform lattice fringes with d-spacing of 0.642 nm, corresponding to the combined WO_3_(010) and (100) planes. The SAED pattern (Fig. 2f) exhibits distinct diffraction rings, validating its crystalline integrity. After thermal treatment at 500 °C, the WO_3_@Co-CoPBA heterostructure (labeled as Co_3_O_4_@WO_3_) undergoes significant structural transformation (Fig. 2g). The sample forms a porous cubic architecture (around 200 nm) with characteristic surface pitting. Notably, high-temperature treatment induces shell-layer decomposition and structural rearrangement, reducing particle size relative to the precursor, while preserving the inner framework's stability and retaining the nano-cube morphology. This core–shell porous heterostructure enables precise modulation of the electronic and chemical environment at active sites, critically influencing the adsorption energy levels of reaction intermediates and enhancing intrinsic catalytic activity. Combined HRTEM (Fig. 2h) and SAED (Fig. 2i) analysis elucidates the hetero-structural interface and further confirms the coexistence of the two phases. The shell layer exhibits lattice fringes of *d* = 0.642 nm (WO_3_(100)), *d* = 0.382 nm (WO_3_(001)), *d* = 0.316 nm (WO_3_(200)), and *d* = 0.167 nm (WO_3_(202)), while the core region displays interplanar spacings of 0.153 nm, 0.197 nm, 0.256 nm, and 0.280 nm corresponding to the (511), (400), (311) and (220) crystal planes of Co_3_O_4_. These results confirm the formation of a strongly coupled core–shell heterostructure between WO_3_ and Co_3_O_4_, where individual crystal structures are preserved while achieving intimate interfacial contact, establishing a structural foundation for efficient electrocatalytic systems.

Energy dispersive spectroscopy (EDS) elemental mapping analysis (Fig. S3) quantitatively characterizes the spatial distribution of C, N, O, Co, and W. Results reveal that C and N are predominantly enriched at the particle surface, while W and O exhibit throughout-particle distribution with uniform coverage of the particle bulk. In contrast, Co accumulates predominantly within the inner core. And this spatial distribution gradient aligns with TEM observations, unambiguously verifying the successful formation of a hetero-structured core–shell architecture in the annealed WO_3_@Co-CoPBA nanoparticles, where a Co_3_O_4_ core is encapsulated by a WO_3_ oxide shell. This structural configuration provides a unique spatial framework for interfacial charge transfer and synergistic effects of active sites, crucial for enhanced electrocatalytic performance.

**Fig. 3:**
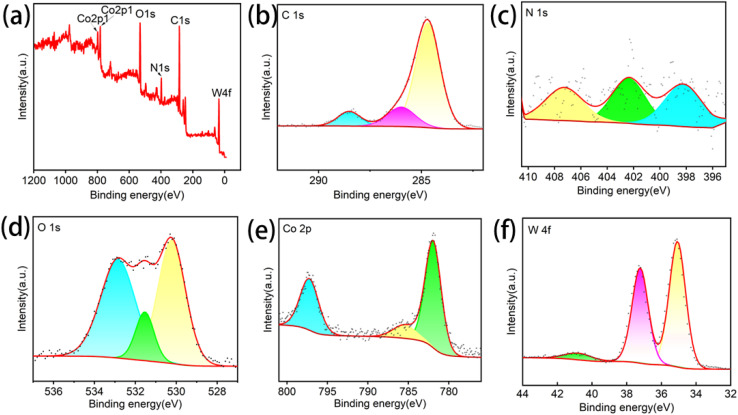
XPS spectrum of WO_3_@Co-CoPBA-500 °C: (a) total spectrum; (b) C 1s; (c)N 1s; (d) O 1s (e) Co 2p; (f) W 4f.

Fig. 3 presents the XPS survey spectrum and elemental valence state analysis of the WO_3_@Co-CoPBA-500 °C heterostructure. The XPS survey (Fig. 3a) detects C, N, O, Co, and W, with spatial distributions consistent with EDS results. The C 1s spectrum (Fig. 3b) exhibits a prominent peak at 284.8 eV corresponding to sp^3^-hybridized carbon (C–C), attributed to lattice distortion from hexagonal ring defects. Weak signals at 285.8 eV (C–OH) and 288.6 eV (HO–C=O) indicate minor surface oxygen-containing functional groups. The N 1s spectrum (Fig. 3c) reveals three distinct peaks at 398.2 eV (pyridinic N), 401.9 eV (graphitic N), and 406.8 eV (N–O), revealing the polytypic nature of nitrogen doping. The O 1s spectrum (Fig. 3d) shows contributions at 530.0 eV (lattice O), 531.5 eV (bridging O), and 531.8 eV (carboxylate O), corresponding to oxygen species in different coordination environments. The Co 2p spectrum (Fig. 3e) features strong peaks at 781.2 eV (Co^3+^ 2p^3/2^) and 796.8 eV (Co^3+^ 2p^1/2^), with a secondary peak at 785.3 eV (Co^2+^ 2p^3/2^), confirming the mixed valence states of Co. The W 4f spectrum (Fig. 3f) displays a doublet at 35.3 eV (W 4f^7/2^) and 37.4 eV (W 4f^5/2^) with a binding energy difference of 2.1 eV, consistent with the characteristic electronic state of W^6+^ in WO_3_. Collectively, these XPS results elucidate the elemental composition and chemical states of the heterostructure, confirmed the coexistence of W^6+^ and Co^3+^/Co^2+^ and the formation of a Co_3_O_4_@WO_3_ hetero-structured core–shell architecture. The observed XPS changes directly support the formation of Co–O–W interfacial bonds and electronic coupling between WO_3_ and Co_3_O_4_ components. The peak shape modifications indicate that interfacial atoms experience different chemical environments compared to bulk atoms, confirming successful heterostructure formation rather than simple physical mixing. This provides critical electronic structural insights into interfacial charge transfer mechanisms and synergistic active site interactions essential for enhanced electrocatalytic performance.

**Fig. 4:**
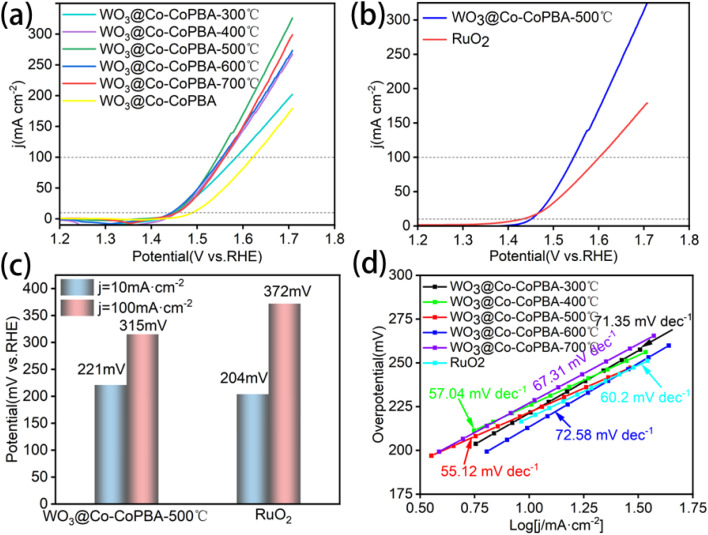
(a) LSV curves for different annealing temperatures; (b) LSV curves for WO_3_@Co-CoPBA-500 °C *versus* RuO_2_; (c) Overpotentials at different current densities; (d) Tafel curves for test samples.

As shown in Fig. 4a, results demonstrate that the 500 °C-annealed sample exhibits the lowest overpotential of 315 mV at 100 mA cm^−2^, significantly outperforming samples annealed at 700 °C (330 mV), 600 °C (325 mV), 400 °C (335 mV), and 300 °C (345 mV). All high-temperature-annealed samples surpass the as-prepared precursor. These results underscore the critical role of thermal treatment in enhancing OER performance.

Comparative analysis with commercial RuO_2_ is shown in Fig. 4b reveals that WO_3_@Co-CoPBA-500 °C exhibits overpotentials of 221 mV at 10 mA cm^−2^ and 315 mV at 100 mA cm^−2^, compared to 204 mV and 372 mV for RuO_2_ (Fig. 4c), while the material shows a slightly higher overpotential than RuO_2_ at low current densities (10 mA cm^−2^), it surpasses commercial benchmarks at high current densities (100 mA cm^−2^). It is noteworthy that RuO_2_ exhibits higher current densities at very high potentials (>1.45 V *vs.* RHE), which may be attributed to different mass transport characteristics and electrode architectures operating in this extreme potential region. However, practical water electrolysis typically operates at current densities of 10–100 mA cm^−2^, where our heterostructure consistently demonstrates superior performance. This enhanced activity is attributed to the three-dimensional porous core–shell architecture formed under an argon atmosphere during high-temperature annealing. The graphene-coated Co_3_O_4_ framework, combined with WO_3_ nanoscale decoration, provides abundant active sites and a hierarchical pore architecture that accelerates mass transport kinetics.

The Tafel analysis was employed to characterize physical parameters, such as the Tafel slope and the exchange current density. This further quantifies reaction kinetics, with fitting results at around 10 mA cm^−2^ yielding Tafel slopes of 71.35 mV dec^−1^ (300 °C), 57.04 mV dec^−1^ (400 °C), 55.12 mV dec^−1^ (500 °C), 72.58 mV dec^−1^ (600 °C), and 67.31 mV dec^−1^ (700 °C) (Fig. 4d). The key to achieving optimal catalytic performance at an annealing temperature of 500 °C lies in three synergistic factors: firstly, this temperature completely converts the Co-CoPBA precursor into a porous Co_3_O_4_ core and forms a graphene encapsulation layer, establishing a strong electronic coupling interface between WO_3_ and Co_3_O_4_; secondly, DFT calculations show that the d-band center shifts towards the Fermi level, and the adsorption energy of OOH* intermediates is optimized based on the Hammer–Nørskov theory to accelerate reaction kinetics; thirdly, the maximum electrochemical active surface area (3.8 cm^2^) and the lowest charge transfer resistance (0.37 Ω) are obtained. In contrast, at 400 °C, the precursor decomposition remains incomplete and the interface coupling is weak, resulting in a higher Tafel slope (57.04 mV dec^−1^); at 600 °C, excessive annealing causes grain aggregation, excessive graphitization, and interfacial stress, resulting in a rebound of the Tafel slope (72.58 mV dec^−1^). Therefore, 500 °C represents the optimal equilibrium point for structural transformation integrity, active site density, interface electronic coupling, and material stability. In addition, the 500 °C-annealed sample exhibits a markedly lower Tafel slope (55.12 mV dec^−1^) than commercial RuO_2_ (60.2 mV dec^−1^), indicating faster charge-transfer kinetics.

Table 1 presents a comprehensive performance comparison between Co_3_O_4_@WO_3_ catalyst and existing Co-based OER catalysts, demonstrating that this work achieves significant breakthroughs across all key metrics. In terms of overpotential, Co_3_O_4_@WO_3_ exhibits excellent performance with 221 mV and 315 mV at 10 mA cm^−2^ and 100 mA cm^−2^, respectively, which are significantly lower than the 280–320 mV and 350–410 mV ranges of comparative catalysts. The Tafel slope of 55.12 mV dec^−1^ is the smallest among all compared materials, indicating the fastest reaction kinetics. More remarkably, the stability performance shows that Co_3_O_4_@WO_3_ exhibits less than 2% performance degradation after 100 hours of continuous operation, far exceeding the typical 20–50 hours lifetime and 5–8% performance loss of other catalysts. This comprehensive performance superiority stems from the unique interface engineering strategy that creates strong electronic coupling effects, optimized active site density (ECSA = 3.8 cm^2^), and exceptional charge transfer efficiency (*R*_ct_ = 0.37 Ω).

**Table 1:** Performance comparison of Co_3_O_4_@WO_3_ with state-of-the-art Co-based OER catalysts

Catalyst	η@10 mA cm^−2^ (mV)	η@100 mA cm^−2^ (mV)	Tafel slope (mV dec^−1^)	Stability	Ref.
Co-MOF/NiO	280	380	67	50 h (5% loss)	34
Co_3_O_4_/rGO	290	350	72	20 h (8% loss)	35
ZIF-67@Co_3_O_4_	310	390	65	30 h (6% loss)	36
Co-PBA/CNT	320	410	78	40 h (7% loss)	37
Co_3_O_4_@WO_3_	221	315	55.12	100 h (<2% loss)	This work

**Fig. 5:**
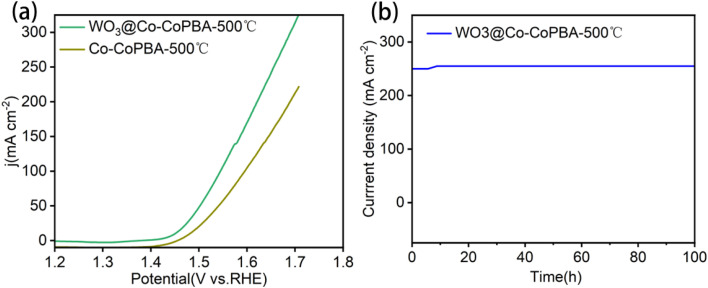
(a) WO_3_@Co-CoPBA-500 °C and Co-CoPBA-500 °C of LSV; (b) stability test.

LSV comparisons between WO_3_@Co-CoPBA-500 °C and Co-CoPBA-500 °C (Fig. 5a) confirm that WO_3_ incorporation significantly enhances OER performance. The WO_3_@Co-CoPBA-500 °C catalyst demonstrates a significant cathodic shift in onset potential and substantially higher current densities across the entire potential range. This improvement arises from increased active site density and elevated specific surface area. Stability tests further demonstrate that the material exhibits no performance degradation over 100 hours of chronoamperometric testing, confirming its exceptional structural stability and electrochemical durability (Fig. 5b). Fig. S4 shows the LSV curve comparison of WO_3_@Co-CoPBA catalyst before and after 100-hours stability testing. The initial overpotential was 315 mV, increasing only to 321 mV after 100 hours, with performance degradation Δ*η* = 1.9% (<2%), demonstrating the catalyst's excellent long-term stability. The two curves almost completely overlap, indicating that the Co_3_O_4_@WO_3_ heterostructure maintained excellent electrocatalytic activity during long-term continuous operation, providing a reliable foundation for practical applications.

Fig. S5 presents the electrochemical active surface area (ECSA) analysis of the WO_3_@Co-CoPBA-500 °C sample. Smooth CV curves were obtained through cyclic voltammetry tests at different scan rates (10–200 mV s^−1^), with current density increasing linearly with scan rate (Fig. S5a). Linear fitting analysis shows a perfect linear relationship between current density and scan rate (*R*^2^ = 0.9999), yielding a double-layer capacitance *C*_dl_ of 152 mF cm^−2^, and subsequently calculating an electrochemical active surface area of 3.8 cm^2^ according to the formula ECSA = *C*_dl_/*C*s (Fig. S5b). Temperature comparison studies demonstrate that within the annealing temperature range of 300–700 °C, the sample annealed at 500 °C exhibits the maximum ECSA value (3.8 cm^2^), confirming that 500 °C is the optimal annealing temperature for achieving superior electrochemical performance (Fig. S5c). This is attributed to the unique hierarchical porous core–shell structure formed at 500 °C, where micropores provide abundant active sites, mesopores enable rapid electrolyte transport, and macropores serve as gas release channels. The interfacial effects of the heterostructure significantly enhance active site accessibility and utilization efficiency compared to single-component materials.

**Fig. 6:**
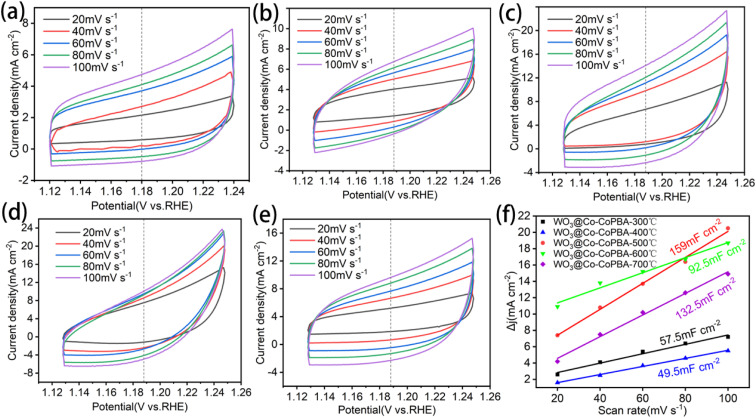
(a)–(e) CV from 300 °C to 700 °C; (f) electrical double-layer capacitance (*C*_dl_) of the test sample.

As shown in Fig. 6, CV curves for WO_3_@Co-CoPBA samples annealed at 300–700 °C (Fig. 6a–e) reveal distinct *C*_dl_ values, determined by the current density difference between the onset and offset of each cycle in the average potential region. The ECSA is calculated through double-layer capacitance (*C*_dl_), which mainly reflects the electrochemically active surface area rather than the total geometric surface area of the material. The temperature-dependent *C*_dl_ trend is summarized in Fig. 6f. Experimental results show *C*_dl_ values of 57.5, 49.5, 159, 92.5, and 132.5 mF cm^−2^ for samples annealed at 300, 400, 500, 600, and 700 °C, respectively. The 500 °C-annealed sample exhibits the highest ECSA, directly correlating with its maximum specific surface area and active site density. Specifically, micropores provide abundant active sites, mesopores promote rapid electrolyte transport, and macropores serve as gas release channels that effectively avoid mass transfer resistance caused by bubble aggregation.

Additionally, we conducted BET specific surface area testing, and the results showed that the BET surface area of WO_3_@Co-CoPBA-500 °C is 60 m^2^ g^−1^ (Fig. S6). The BET surface area reflects the total physical surface area, while ECSA reflects the electrochemically active surface area. The ratio between these two parameters helps evaluate the utilization efficiency of active sites, and the ratio is reasonable, indicating good active site exposure in the material. Furthermore, based on ECSA analysis, pore structure characterization, and BET data, we further elucidated the mechanism by which pore structure enhances catalytic activity through multiple pathways: enhancing mass transfer kinetics, optimizing gas release channels, and improving active site accessibility.

Equivalent circuit model fitting of EIS data reveals charge transfer resistance (*R*_ct_) values of 2.07 Ω, 1.21 Ω, 0.37 Ω, 0.63 Ω, and 0.43 Ω for WO_3_@Co-CoPBA samples annealed at 300–700 °C, respectively (Fig. S7). The 500 °C-annealed sample exhibits the lowest *R*_ct_, indicating optimal electrocatalytic activity and fastest charge transfer kinetics. This confirms the synergistic interaction between WO_3_ and Co-CoPBA and the synergistic enhancement of catalytic kinetics by the annealing process, which significantly improves interfacial charge transfer efficiency and electrochemical reaction kinetics.

Fig. S8 presents the Nyquist plots of WO_3_@Co-CoPBA at different annealing temperatures (300–700 °C), clearly reflecting the significant influence of annealing temperature on charge transfer resistance (*R*_ct_). The diameters of the five semicircular curves in the figure directly correspond to their respective *R*_ct_ values, where the 300 °C sample exhibits the largest semicircle (*R*_ct_ = 2.07 Ω), while the 500 °C annealed sample (labeled as optimal) shows the smallest semicircle (*R*_ct_ = 0.37 Ω), indicating its optimal charge transfer efficiency. In comparison, the charge transfer resistances of the 400 °C (1.21 Ω), 600 °C (0.63 Ω), and 700 °C (0.43 Ω) samples are all significantly higher than that of the 500 °C sample. This result convincingly demonstrates that 500 °C annealing treatment can achieve a strong electronic coupling interface between WO_3_ and Co_3_O_4_, forming optimal charge transport pathways and thereby obtaining superior electrocatalytic performance.

**Fig. 7:**
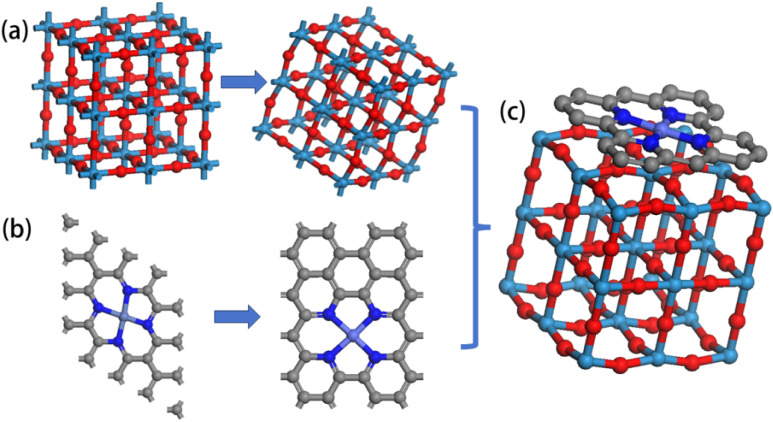
(a) WO_3_ and its optimized structure; (b) carbon-encapsulated Co_3_O_4_ and its optimized structure; (c) WO_3_@Co-CoPBA-500 °C molecular model.

The model development involved three stages: (1) structural optimization of WO_3_ and Co_3_O_4_ crystals; (2) embedding of Co_3_O_4_ within a carbon substrate; and (3) heterojunction re-optimization to achieve an energy-converged stable structure, as shown in Fig. 7. For the slab model construction, we built periodic surface structures based on the experimentally determined crystal orientations from our HRTEM analysis. The WO_3_ component was modeled using the most stable (001) surface, while the Co_3_O_4_ was represented by the (311) surface. The slab models consisted of 6–8 atomic layers with the bottom two layers fixed during optimization to simulate bulk behavior, while the top layers were allowed to relax. The calculations employed a *k*-point mesh of 3 × 3 × 1 for surface sampling with an energy cutoff of 450 eV. The heterojunction interface was constructed by placing the WO_3_ slab in contact with the carbon-encapsulated Co_3_O_4_ surface, with careful attention to lattice matching and interfacial strain minimization. A vacuum layer of 15 Å was applied in the direction perpendicular to the surface. Convergence criteria of 0.02 eV Å^−1^ for forces and 10^−5^ eV for energy differences were applied during geometric optimizations. Spin-polarized calculations were performed to account for the magnetic properties of Co_3_O_4_. The Gibbs free energy calculations for OER intermediates followed the computational hydrogen electrode model, where Δ*G* = Δ*E* + Δ*ZPE* − *T*Δ*S* + e*U*.

DFT calculations and experimental observations consistently demonstrate that the Co_3_O_4_@WO_3_ heterostructure exhibits significantly enhanced alkaline stability compared to pure Co_3_O_4_. The strong electronic coupling at the heterointerface effectively suppresses the dissolution of active materials, which is experimentally confirmed by the minimal performance decay (<2%) observed during our 100-hours testing. Pure Co_3_O_4_-based catalysts typically exhibit obvious degradation within shorter periods (<50 hours) under similar alkaline conditions due to metal ion leaching. The WO_3_ component in our heterostructure not only provides inherent chemical stability, but its shell structure also offers additional protection for the internal Co_3_O_4_ active sites while maintaining high OER activity through interfacial electronic modulation. This design strategy provides an effective approach for achieving a balance between high activity and high stability.

**Fig. 8:**
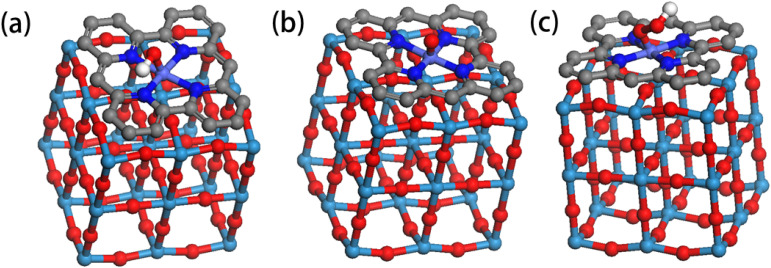
Model of adsorption process (a) O*; (b) OH*; (c) OOH*.

**Fig. 9:**
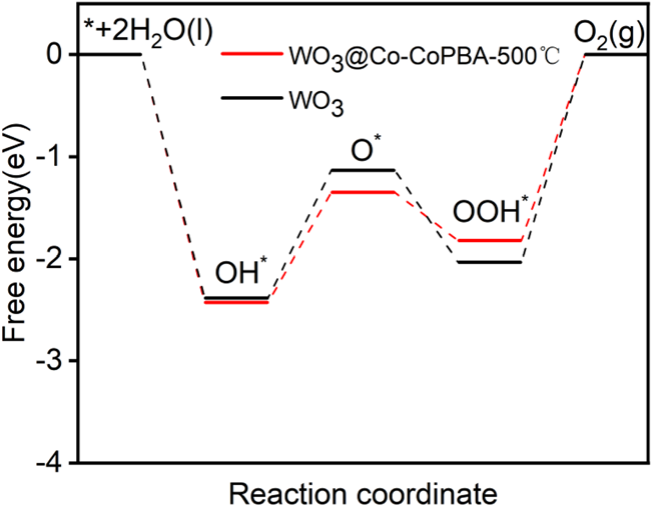
Overpotential step diagram of WO_3_ and WO_3_@Co-CoPBA-500 °C OER.

OER proceeds *via* four elementary reaction steps. Reaction pathway modeling (Fig. 8) enabled calculation of Gibbs free energy changes (Δ*G*) for each step. By aligning these values with the thermodynamic reference potential (1.23 V *vs.* RHE), the overpotential *η* associated with each step was derived. As shown in the reaction energy diagram (Fig. 9), exothermic steps (Δ*G* < 0) represent the spontaneous tendencies, while the rate-determining step corresponds to the endothermic process with the highest energy barrier. Analysis reveals that the WO_3_@Co-CoPBA-500 °C system exhibits significantly lower maximum overpotential compared to reference systems, indicating enhanced OER reactivity. The active site configuration was determined by systematically examining different adsorption sites at the CO_3_O_4_@WO_3_ interface, including both individual metal centers and interfacial bridge sites. The calculations focused on the adsorption energies of key OER intermediates (O*, OH*, and OOH*), with particular attention to identifying the rate-determining step. The DFT calculations provide quantitative evidence supporting Co sites as active centers. The adsorption energies of key OER intermediates on Co sites are as follows: OH* (−0.87 eV), O* (−1.23 eV), and OOH* (−0.45 eV). Importantly, the formation of the WO_3_@Co_3_O_4_ heterointerface shifts the d-band center of Co sites from −2.1 eV (pure Co_3_O_4_) to −1.9 eV (heterostructure), representing a 0.2 eV upward shift toward the Fermi level. This shift optimizes the adsorption Gibbs free energy of the rate-determining OOH* step from 1.82 eV to 1.67 eV, directly explaining the enhanced catalytic activity observed experimentally. In contrast, calculations on W sites show much weaker interactions with OER intermediates and no significant involvement in the catalytic cycle, confirming their role as electronic modulators rather than direct active sites.

**Fig. 10:**
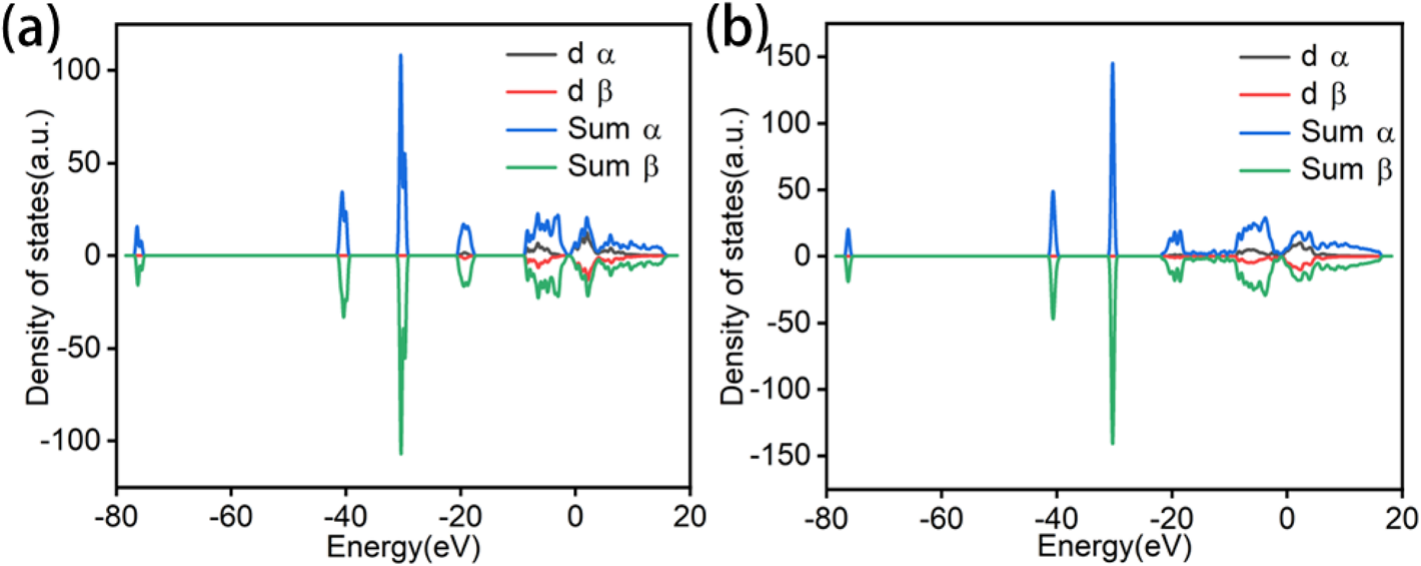
Total DOS and d-band center of (a) WO_3_ and (b) WO_3_@Co-CoPBA-500.

Comparative DOS analysis (Fig. 10) between WO_3_ and WO_3_@Co-CoPBA-500 °C reveals that the d-band center of WO_3_ lies deeper below the Fermi level, whereas the d-band center in the heterostructure shifts closer to the Fermi level. Specifically, the d-band center of pure WO_3_ is located approximately 2–3 eV below the Fermi level, while in the Co_3_O_4_@WO_3_ heterostructure, the d-band center shifts upward by approximately 0.5–0.8 eV. This upward shift is a critical indicator of enhanced electronic coupling and improved reactivity, as it increases the antibonding states' occupancy and strengthens the interaction between metal d-orbitals and adsorbate molecular orbitals according to the d-band theory established by Hammer and Nørskov. The d-band center modulation directly correlates with the optimization of adsorption Gibbs free energy for OER intermediates. Our calculations show that the Co_3_O_4_@WO_3_ heterostructure exhibits substantially reduced energy barriers compared to pure WO_3_, with the rate-determining step (typically *O → *OOH formation) showing improved thermodynamic favorability. The density of states (DOS) analysis revealed that the d-band center shifts closer to the Fermi level in the heterostructure compared to pure WO_3_, suggesting that the metallic sites are more favorable for adsorption and activation of reaction intermediates, thereby enhancing interfacial charge transfer efficiency and intrinsic catalytic activity.

Based on the observed d-band center shifts and enhanced electronic coupling evidenced by XPS analysis, our heterostructure may potentially form a type II (staggered gap) heterojunction that facilitates charge separation. The XPS results (Fig. 3) show complementary binding energy shifts: Co 2p peaks shift positively (781.2 eV for Co^3+^) while W 4f peaks shift negatively (35.3 eV), indicating electron transfer from Co to W sites. This charge redistribution pattern is characteristic of type II band alignment where electrons and holes are spatially separated across the interface. Additionally, DFT calculations reveal a 0.2 eV upward shift of the Co d-band center toward the Fermi level, confirming substantial electronic restructuring. The formation of a type II heterojunction would explain the enhanced OER performance through efficient charge separation, which prevents electron–hole recombination and optimizes the adsorption energy of OER intermediates (OOH* formation energy reduced from 1.82 eV to 1.67 eV), ultimately resulting in superior catalytic activity.

## 4 Conclusion

This work introduces several key innovations: (1) the use of Co-CoPBA precursors for topological reconstruction, creating unprecedented electronic coupling interfaces; (2) achievement of superior OER performance (*η* = 315 mV @ 100 mA cm^−2^) that outperforms both commercial RuO_2_ and most reported Co-based catalysts; (3) exceptional stability (100 h with <2% degradation) that significantly exceeds the typical 20–50 h lifetime of similar materials; and (4) comprehensive theoretical understanding through d-band center engineering. In summary, we have developed a simple and novel strategy to synthesize Co_3_O_4_@WO_3_ hetero-structured heterostructures with cost-effective and high-performance alkaline OER activity through hydrothermal synthesis of WO_3_ on Co-CoPBA precursors coupled with annealing temperature modulation. The superior OER performance is attributed to the synergistic effects between Co active sites and WO_3_ electronic modulators. Specifically, Co_3_O_4_ provides abundant Co^3+^/Co^2+^ active sites that serve as the primary catalytic centers for OER, while WO_3_ nanorods modulate the electronic structure of these Co sites through interfacial electronic coupling, simultaneously providing high conductivity and structural stability. Experimental and DFT calculation results revealed that the 500 °C thermal treatment induces topological reconstruction of Co-CoPBA into a porous Co_3_O_4_/graphene heterostructure, synergistically interfacing with WO_3_ to enhance active site density (ECSA = 3.8 cm^2^) and electron transfer efficiency (Tafel slope = 55.12 mV dec^−1^). The material exhibits an overpotential of 315 mV at 100 mA cm^−2^, outperforming commercial RuO_2_(372 mV). We expect that these initial studies on Co_3_O_4_@WO_3_ heterostructures will provide access to unprecedented multifunctional materials for the development of high-performance energy storage and conversion devices.

## Conflicts of interest

All authors disclosed no relevant relationships.

## Supplementary Material

RA-015-D5RA04599A-s001

## Data Availability

The authors confirm that the data supporting the findings of this study are available within the article and as its SI materials. See DOI: https://doi.org/10.1039/d5ra04599a.

## References

[cit1] Huang H., Xue Q., Zhang Y. (2019). et al.. Electrochim. Acta.

[cit2] Li Y. D., Chen B. J., Zhang H. M. (2021). et al.. ChemElectroChem.

[cit3] Song M., Bai Y., Li J., Qi X. (2025). RSC Adv..

[cit4] Wu T., Sun M. Z., Huang B. L. (2022). Rare Met..

[cit5] Wu Z. P., Lu X. F., Zang S. Q., Lou X. W. (2020). Adv. Funct. Mater..

[cit6] Wu H., Feng C., Zhang L., Zhang J., Wilkinson D. P. (2021). Electrocatalysis.

[cit7] P V., Liu X. H., Babu R. R. (2023). et al.. Chemosphere.

[cit8] Periyasamy V., Liu S. H., Sathiya M. (2024). et al.. ACS Appl. Nano Mater..

[cit9] Cao L. M., Hu Y. W., Tang S. F. (2018). et al.. Adv. Sci..

[cit10] Li B., Sun L., Bian J. (2020). et al.. Appl. Catal., B.

[cit11] Khan M. E., Khan M. M., Cho M. H. (2016). RSC Adv..

[cit12] Chen J., Chen C., Qin M. (2022). et al.. Nat. Commun..

[cit13] Xu X., Xiao Y., Zhao R. (2025). et al.. Ceram. Int..

[cit14] Lu K., Wang Z., Wu Y. (2023). et al.. Chem. Eng. J..

[cit15] Dhanya A. R., Haridoss P., Ramaprabhu S. (2024). Int. J. Hydrogen Energy.

[cit16] Zheng Y., Cao L., Xing G. (2019). et al.. RSC Adv..

[cit17] Mane P., Burungale V., Bae H. (2024). et al.. Renewable Sustainable Energy Rev..

[cit18] Zhang J., Zhu G., Liu W. (2020). et al.. J. Alloys Compd..

[cit19] Gondal M. A., Hameed A., Yamani Z. H., Suwaiyan A. (2004). Chem. Phys. Lett..

[cit20] Phiankoh S., Prajongtat P., Chareonpanich M. (2020). et al.. Energy Technol..

[cit21] Ye L., Wen Z. (2019). Int. J. Hydrogen Energy.

[cit22] Stock N., Biswas S. (2012). Chem. Rev..

[cit23] Yaqoob L., Noor T., Iqbal N. (2021). et al.. J. Alloys Compd..

[cit24] Xu W., Tao Y., Zhang H. (2024). et al.. Small.

[cit25] Tang S., Li L., Ren H. (2019). et al.. Mater. Today Chem..

[cit26] Tan Y., Chen D., Kotsiubynskyi V. (2025). et al.. J. Energy Chem..

[cit27] Sun W., Wei Z., Qi J. (2021). et al.. Chin. J. Chem..

[cit28] Zhang X., Khan I. U., Huo S. (2020). et al.. Electrochim. Acta.

[cit29] Jiang M., Fan X., Cao S. (2021). et al.. J. Mater. Chem. A.

[cit30] Aravind M., Kumaresubitha T., Ahmed N., Velusamy P. (2022). Inorg. Chem. Commun..

[cit31] Jin S. (2019). ACS Energy Lett..

[cit32] Surendhar S., Paramasivam S., Chemie P. V. (2024). et al.. Z. Phys. Chem..

[cit33] Velusamy P., Babu R. R., Sathiya M. (2022). et al.. New J. Chem..

[cit34] Zeng X., Cai Z., Zhang C. (2022). et al.. Mater. Res. Lett..

[cit35] Wu Z., Sun L. P., Yang M. (2016). et al.. J. Mater. Chem. A.

[cit36] Li X., You S., Du J. (2019). et al.. J. Mater. Chem. A.

[cit37] Lin Y. C., Chuang C. H., Hsiao L. Y. (2020). et al.. ACS Appl. Mater. Interfaces.

