# Supplementary material for: A cost-effective Co3O4@WO3 hetero-structure derived from WO3@Co-CoPBA for oxygen evolution reaction
Source: RSC Adv. 2025 Aug 22;15(36):29925–36. doi: 10.1039/d5ra04599a (PMC12376896; doi:10.1039/d5ra04599a)
Supplement: RA-015-D5RA04599A-s001 [file RA-015-D5RA04599A-s001.pdf]

Supplementary Information (SI) for RSC Advances.
This journal is © The Royal Society of Chemistry 2025

A cost-effective $WO_3@Co_3O_4$ hetero-structure derived from WO_3@Co-CoPBA for oxygen evolution reaction

Zhenwei Yan[a]*, Zihao Wei[a], Zhaojun Tan[a], Shuaihui Guo[a], Zhipeng Fang[a], Wen Wang[a], Gang Li[a], Xianjie Yuan[a], Mingqi Tang[b], Zaiqiang Feng[b]

a School of Mechanical Engineering, North China University of Water Resources and Electric Power, Zhengzhou 450011, PR China

b School of Materials Science and Engineering, North China University of Water Resources and Electric Power, Zhengzhou 450011, PR China

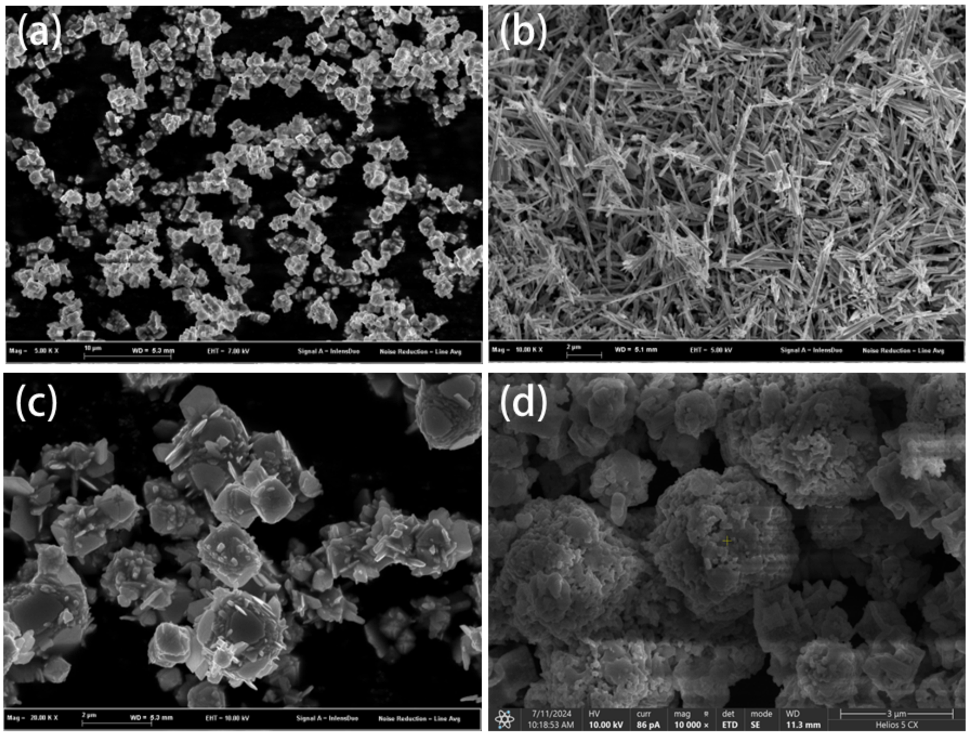

Figure S1. Scanning electron microscopy (SEM) images: (a)Co-CoPBA cube; (b)WO_3 nanorods; (c)WO_3@Co-CoPBA; (d)WO_3@Co-CoPBA-500°C.

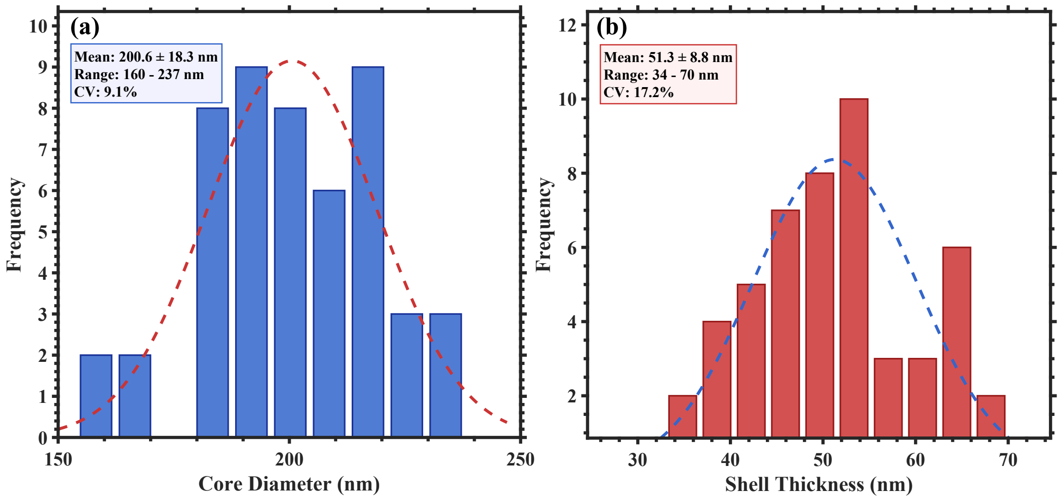

Figure S2. Statistical analysis of core-shell morphology for WO_3@CO-COPBA heterostructure. (a) Core diameter distribution; (b) Shell thickness distribution.

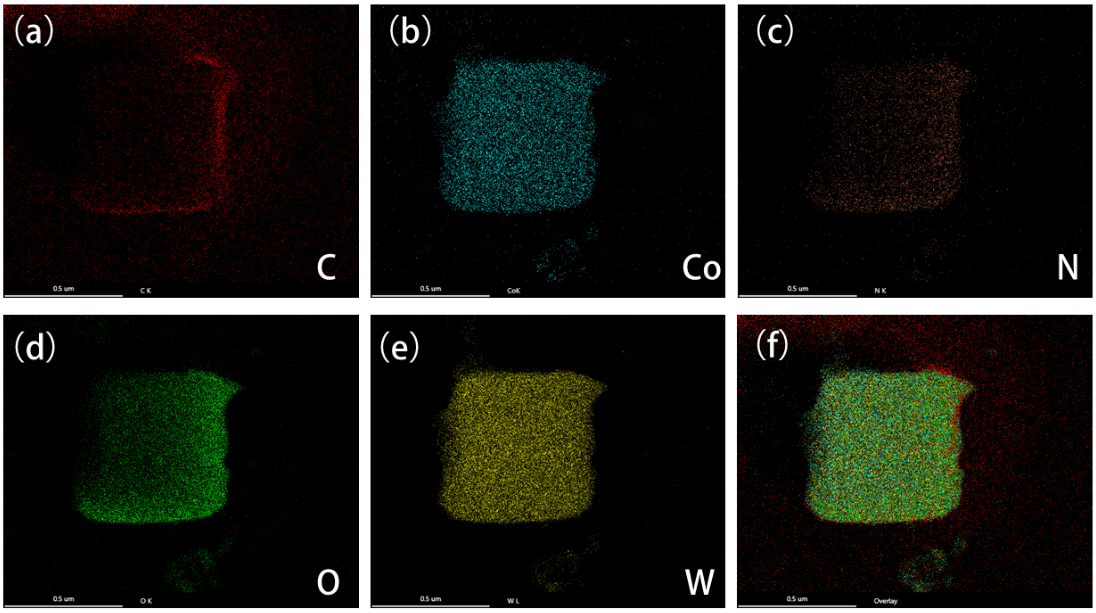

Figure S3. Energy-dispersive spectroscopy (EDS) map of WO_3@Co-CoPBA (a) C; (b) Co; (c) N; (d) O; (e) W; (f) Total spectrum.

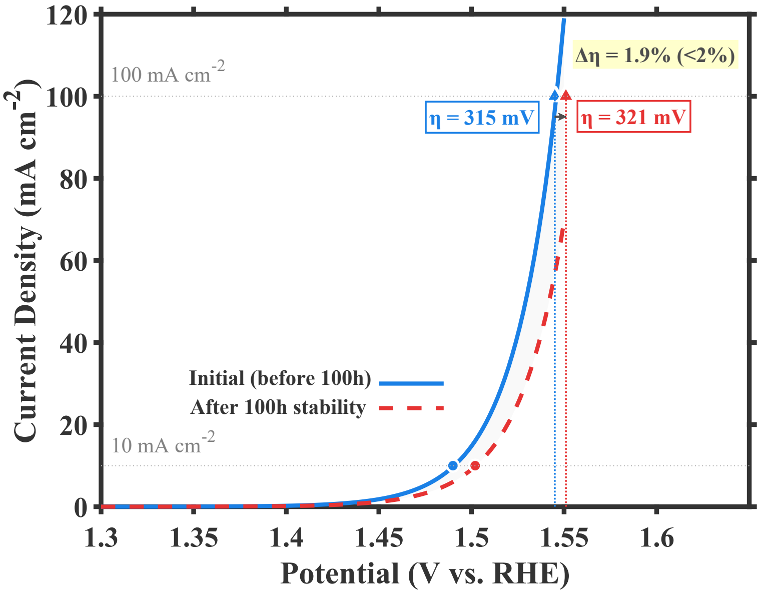

Figure S4. LSV curves before and after 100h stability test.

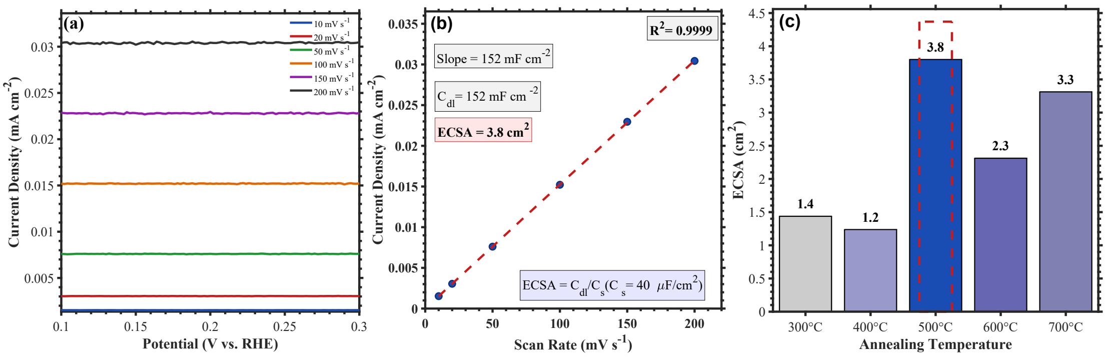

Figure S5. ECSA analysis of WO_3@Co-CoPBA-500°C. (a) Cyclic voltammetry curves; (b) Linear fitting for C_{dl}; (c) Temperature influence.

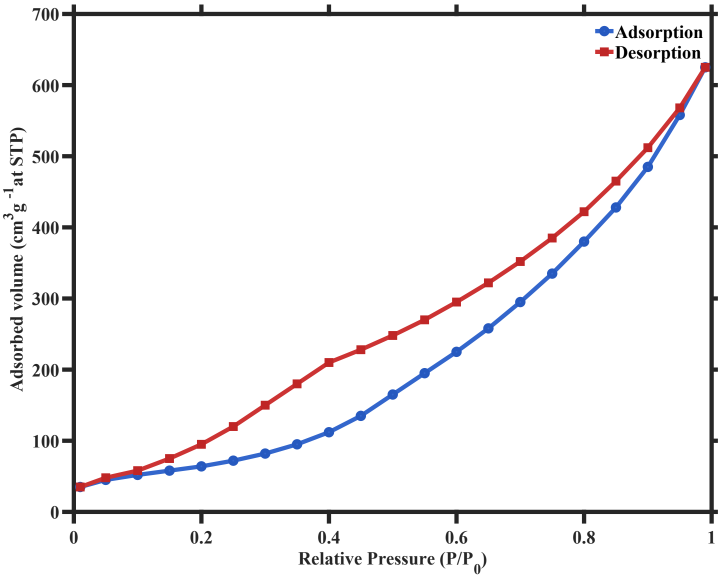

Figure S6. The specific surface area of WO_3@Co-CoPBA-500℃.

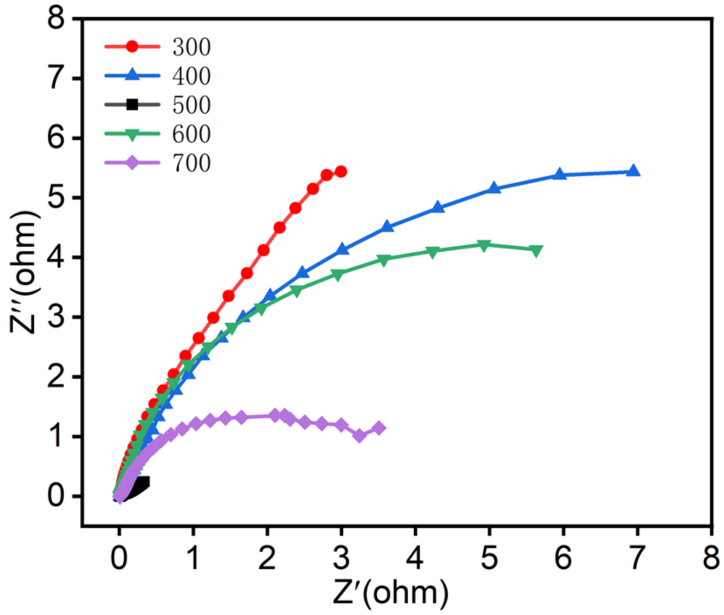

Figure S7. Electrochemical impedance spectroscopy (EIS) of WO_3@Co-CoPBA 300℃–500℃.

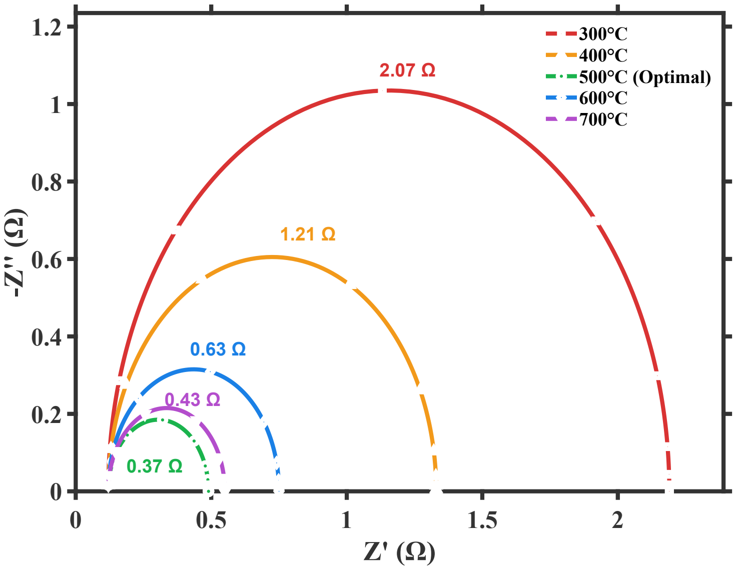

Figure S8. Nyquist plots of WO_3@Co-CoPBA at different annealing temperatures.
